# Epidemiologic Research on Malformations Associated with Cleft Lip and Cleft Palate in Japan

**DOI:** 10.1371/journal.pone.0149773

**Published:** 2016-02-22

**Authors:** Hiroshi Koga, Koichi Iida, Tomoki Maeda, Mizuho Takahashi, Naoki Fukushima, Terufumi Goshi

**Affiliations:** 1 Department of Pediatrics, National Hospital Organization Beppu Medical Center, Oita, Japan; 2 Department of Neonatology, Oita Prefectural Hospital, Oita, Japan; 3 Department of Pediatrics, Oita University Faculty of Medicine, Oita, Japan; 4 Department of Pediatrics, Almeida Hospital, Oita, Japan; 5 Department of Pediatrics, Nakatsu Municipal Hospital, Oita, Japan; Tabriz University of Medical Sciences, ISLAMIC REPUBLIC OF IRAN

## Abstract

To investigate malformations associated with cleft lip and cleft palate, we conducted surveys at neonatal intensive care units (NICUs) and other non-NICU facilities and to determine whether there are differences among facilities. The regional survey investigated NICU facilities located in Oita Prefecture, including 92 patients with cleft lip and palate (CLP) or cleft palate (CP) that occurred between 2004 and 2013, and the national survey investigated oral surgery, plastic surgery, and obstetrics and gynecology facilities located in Japan, including 16,452 patients with cleft lip (CL), CLP, or CP that occurred since 2000. The incidence per 10,000 births was 4.2, 6.2, and 2.8 for CL, CLP, and CP, respectively, according to the national survey, and 6.3 and 2.9 for CLP and CP, respectively according to the regional survey. These results indicated comparable incidences between the two surveys. In contrast, when the survey results on malformations associated with CLP and CP according to the ICD-10 classification were compared between the national survey conducted at oral surgery or plastic surgery facilities and the regional survey conducted at NICU facilities, the occurrence of associated malformations was 19.8% vs. 41.3% for any types of associated malformation, 6.8% vs. 21.7% for congenital heart disease, and 0.5% vs. 16.3% for chromosomal abnormalities. These results indicated that the incidences of all of these associated malformations were significantly greater in the survey conducted at NICU facilities and similar to the findings from international epidemiological surveys. When comparing the survey conducted at obstetrics facilities vs. NICU facilities, the occurrence of associated malformations was similar results as above. The incidence of CLP and CP was not different between surveys conducted at NICU facilities vs. non-NICU facilities; however, when conducting surveys on associated malformations, it is possible to obtain accurate epidemiological data by investigating NICU facilities where detailed examinations are thoroughly performed.

## Introduction

Cleft lip and cleft palate, namely cleft lip (CL), cleft lip and palate (CLP), and cleft palate (CP), are major congenital malformations diagnosed at birth; they occur at a rate of approximately one in 700 births.[1] Infants affected with cleft lip and cleft palate require multidisciplinary treatment from birth, and especially in CLP and CP, infants are transferred from the obstetrics to the neonatal intensive care unit (NICU) shortly after birth in Japan, since specialized feeding support is necessary. Previous epidemiological surveys in Japan on cleft lip and cleft palate were conducted at oral surgery and obstetrics facilities. Thus, it is possible that these surveys did not include severely affected infants who died early, or that the associated malformations of the affected infants were not accurately diagnosed in these surveys.[2] In the present study, a regional epidemiological survey obtaining accurate data on associated malformations was performed in NICU facilities, where affected infants are admitted and undergo detailed examinations. Furthermore, epidemiological data on cleft lip and cleft palate that occurred nationwide in Japan were also collected. The Japanese nationwide survey results are reported together with the regional results.

## Methods

### 1. Regional survey

#### Data collection

Oita Prefecture, with a population of approximately 1 million and annual births of approximately 10,000, is located in the southern region of Japan. This study and the opt-out consent method was approved by the Institutional Revew Boards of five facilities in Oita Prefecture, including the National Hospital Organization Beppu Medical Center, Oita Prefectural Hospital, Oita University Faculty of Medicine, Almeida Hospital, and Nakatsu Municipal Hospital. All medical data from the participants were anonymized and compiled at the Beppu Medical Center, and it was therefore considered that there was no risk that individual participating patients would be identified. The study plan and the choice to freely refuse participation were announced through the hospital bulletin at the five facilities. Patients were considered to consent to the study if there was no request to refuse participation. Clinical information was collected from all neonates with CLP or CP who were admitted to the NICU at the above five facilities in Oita Prefecture between January 2004 and December 2013. Data for the following clinical information were extracted: cleft type, sex, family history, abnormalities during pregnancy, prenatal ultrasound screening test results, gestational age, Apgar score, birth weight, presence or absence of associated malformations and their details, neonatal feeding method, infant death, age at initial CP repair, remaining postoperative symptoms, and postoperative physical development. Associated malformations were classified according to International Classification of Diseases, 10^th^ edition (ICD-10).[3] The z-scores of body weight and height were calculated using the 2000 Infant Physical Development Survey by the Ministry of Health, Labour and Welfare and the School Health Statistics Research data by the Ministry of Education, Culture, Sports, Science and Technology, and long-term physical development was evaluated.

### 2. National survey

#### Data collection

To compare with our regional survey results, Japanese survey reports related to cleft lip and cleft palate were aggregated. Reports were searched using the Japan Medical Abstracts Society database. Inclusion criteria were: 1) surveys published since 2000 that investigated patients with cleft lip and cleft palate, and 2) clinical statistical studies conducted in a specific region or medical institutions in Japan. Exclusion criteria were: 1) survey reports with overlap of study patients, 2) surveys that did not include all three disorders (CL, CLP, and CP), and 3) surveys that did not describe the patients’ clinical information. The search keywords “cleft lip,” “cleft lip and palate,” or “cleft palate” were used. This resulted in 3398 hits, of which 62 met the inclusion criteria. After excluding 28 reports that met the exclusion criteria, 34 reports were ultimately included in the present study.

### 3. Statistical analysis

The incidences of CLP and CP were calculated based on the number of live births (99,704) in Oita Prefecture during a 10-year period. Data are expressed as the actual numbers (percentage) or medians (range). For statistical analysis, the chi-square test or Fisher’s exact test was used for nominal variables, and the Mann-Whitney U test was used for continuous variables. All statistical analyses were conducted using SPSS statistics version 20 (IBM, Armonk, NY). P<0.05 was considered significant. The first author takes complete responsibility for the integrity of the data and the accuracy of the data analysis.

## Results

### 1. Regional survey

During the survey period from January 2004 to December 2013, 63 patients with CLP and 29 patients with CP were identified. None of these patients refused to participate in the study. During this period, the total number of live births in Oita Prefecture was 99,704, resulting in CLP and CP incidences at birth of 6.3/10,000 births (0.06%) and 2.9/10,000 births (0.03%), respectively. Of the 63 CLP patients, 42 were boys and 21 were girls (male: female = 2:1), and 45 (71%) had a unilateral cleft, which occurred more commonly on the left side (n = 26, 58%) (Table 1). Of the 29 CP patients, 6 were boys and 23 were girls (male:female≈1:4).

**Table 1:** Distribution of cleft pattern by cleft type and sex in a regional study.

	Cleft type			Sex		Total
				Male	Female	
**CLP**	Unilateral	Left	26	18	8	45
		Right	19	12	7	
		(L: R = 1.4: 1)		(M:F = 2:1)		
	Bilateral			12	6	18
				(M:F = 2:1)		
**CP**	Complete		18	6	23	18
	Incomplete		11	(M:F = 1:4)		11
				48	44	92

Basic clinical information of the study patients during the perinatal period is shown in Table 2. A family history of cleft lip and cleft palate was present in 9 of 92 patients (10%). Abnormalities during pregnancy were observed in 37 of 92 patients (40%), and impaired fetal growth and abnormal amniotic fluid volume accounted for 89% of such abnormalities. A prenatal ultrasound screening test detected abnormalities in 38 of 92 patients (41%), and while CL was detected in 29 of 63 CLP patients (46%), CP was only detected in 1 of 29 CP patients (3%) (p = 0.007). A significantly greater percentage of CP patients had a low birth weight (<2,500 g) compared to CLP patients (p = 0.04). Moreover, the percentage of patients with neonatal asphyxia (Apgar score <7) was greater in CP than in CLP, and a significant difference was observed between the two groups of patients in the Apgar score measured 5 minutes after birth (p = 0.035). Malformations associated with cleft lip and cleft palate occurred in 41.3% of all patients, and they occurred significantly more frequently in those with CP (p<0.001).

**Table 2:** Perinatal baseline characteristics in a regional study.

	CLP	CP	P Value
	n = 63 (%)	n = 29 (%)	
**Family history of orofacial cleft**	4 (6)	5 (17)	0.134
**Problem during pregnancy**	22 (35)	15 (52)	0.170
Fetal growth restriction	10	10	
Polyhydramnios	5	3	
Oligohydramnios	4	1	
Pregnancy-induced hypertension	3	1	
**Prenatal ultrasonographic diagnosis**	32 (51)	6 (21)	0.007
Orofacial cleft alone	25	1	
Orofacial cleft with other defects	4	0	
Other defects alone	3	5	
**Gestational age (weeks)**			0.444
< 36	6 (10)	7 (24)	
37–38	29	9	
39–40	22	9	
> 40	6	4	
**Birth weight (g)**			0.040
< 2500	16 (25)	15 (52)	
2500–3000	26	8	
3000–3500	19	5	
> 3500	2	1	
**Apgar score at 1 min**			0.088
< 3	4	3	
4–7	12	8	
> 8	47 (75)	18 (62)	
**Apgar score at 5 min**			0.035
< 3	1	1	
4–7	4	8	
> 8	58 (92)	20 (69)	
**Associated congenital malformation**	18 (29)	20 (69)	< 0.001

A list of associated malformations according to ICD-10 classification is shown in Table 3. Congenital heart disease was the most common disease category (n = 20), followed by a chromosomal abnormality. Ventricular septal defect and trisomy 13 were the most common single diseases (n = 8 each).

**Table 3:** Associated congenital malformations in a regional study.

	CLP	CP	Total
	(Number of cases)*	(Number of cases)*	n = 38 (%)
**Nervous system**	Horoprosencephaly (2)		3 (8)
	Schizencephaly (1)		
**Eye, ear, face and neck**	Persistent pupillary membrane (1)	Microphthalmia (1)	2 (5)
**Circulatory system**	Ventricular septal defect (2)	Ventricular septal defect (6)	20 (53)
	Double outlet right ventricle (2)	Double outlet right ventricle (3)	
	Atrioventricular septal defect (1)	Atrioventricular septal defect (1)	
	Patent ductus arteriosus (1)	Patent ductus arteriosus (1)	
	Transposition of the great arteries (1)	Hypoplastic left heart syndrome (1)	
		Tetralogy of Fallot (1)	
**Respiratory system**	Pulmonary hypoplasia (1)		1 (3)
**Other digestive system**	Imperforate anus (1)	Esophageal atresia (1)	2 (5)
**Genital organs**	Hypospadias (1)	Hypospadias (1)	4 (11)
	Hypoplasia of penis (1)		
	Pseudohermaphroiditism (1)		
**Urinary system**	Hydronephrosis (1)	Renal dysplasia (1)	2 (5)
**Musculoskeletal system**	Arthrogryposis multiplex congenita (1)	Arthrogryposis multiplex congenita (1)	6 (16)
	Congenital diaphragmatic hernia (1)	Hypochondrogenesis (1)	
		Spondyloepiphyseal dysplasia (1)	
		Camptodactyly (1)	
**Other congenital malformation**	CHARGE association (1)	Stickler syndrome (3)	8 (21)
	Cornelia de Lange syndrome (1)	Pierre-Robin syndrome (2)	
		Cayler syndrome (1)	
**Chromosomal abnormality**	Trisomy 13 (4)	Trisomy 13 (4)	15 (39)
	Unbalanced translocation (2)	Unbalanced translocation (2)	
	Trisomy 18 (1)	Karyotype 49, XXXXY (2)	

* Each applicable type of condition was counted (multiples items allowed per patient).

The patients’ clinical courses after birth are shown in Table 4. Oral feeding using a Hotz plate was the most common (42/92 patients, 46%) primary feeding method during the neonatal period. Tube feeding was used in 25 of 92 patients (27%) due to difficulties in oral feeding. Of the 25 patients requiring tube feeding, 21 (84%) presented with some type of associated malformation. Five of 92 patients (3%) primarily received parenteral nutrition due to serious complications; all five of these patients died during infancy. Thirteen of 92 patients (14%) died during infancy, and 10 of these 13 patients (77%) also presented with either congenital heart disease or a chromosomal abnormality. The timing of the initial cleft palate repair could be verified in 35 CLP patients and 8 CP patients, excluding those who died during infancy, and this repair procedure was performed at a median age of 1.8 years (1.2 to 6.4) and 1.7 years (1.4 to 5.1), respectively. Commonly observed remaining postoperative symptoms were otitis media (n = 18, 20%) and hearing loss (n = 9, 12%). Malocclusion was evaluated when the patient was at least 5 years old and was detected in 5 patients. Speech impairment was evaluated when the patient was at least 3 years old and was detected in 3 patients. Physical measurements conducted at ≥1 year of age could be verified in 39 CLP patients and 11 CP patients, excluding those who had died during infancy. The z-scores for body weight were -0.04 (-7.22 to 1.87) for CLP and -0.35 (-5.43 to 1.63) for CP, and the z-scores for height were -1.30 (-5.76 to 2.13) for CLP and -0.72 (-6.35 to 0.98) for CP. When comparisons were made between the presence and absence of associated malformation, the z-scores for body weight were -1.33 (-7.22 to 1.63) with associated malformations vs. 0.00 (-2.10 to 1.87) without associated malformations (p = 0.124), and the z-scores for height were -1.34 (-6.35 to 2.13) with associated malformations vs. -0.55 (-2.80 to 1.67) without associated malformations (p = 0.121), indicating a tendency for patients to have a growth disorder when an associated malformation was present, although neither z-score reached significance.

**Table 4:** Clinical management and physical growth in a regional study.

	CLP	CP	P Value
	n = 63 (%)	n = 29 (%)	
**Main feeding method during the neonatal period**			
Usual oral feeding	5	7	
Bottle feeding with cleft palate nipple	5	1	
Oral feeding with Hotz-type plate	40 (63)	4	
Tube feeding	10	15 (52)	
Parenteral nutrition	3	2	
**Infant death**	6 (10)	7 (24)	0.103
**Age at surgical repair of hard palate (years)**			0.795
1–1.4	4	1	
1.5–2	24 (69)	5 (43)	
> 2	7	2	
**Postoperative dysfunction and illness**			
Otitis media	14	4	
Hearing impairment	6	5	
Malocclusion	5	0	
Speech difficulty	3	0	
Feeding disorder	0	1	
**Age at body measurement (years)**			0.062
1–3	30 (77)	8 (73)	
4–6	7	2	
> 7	2	1	
**Weight z-score**			0.806
< -2	7	3	
-2–0	13	3	
> 0	19 (49)	5 (45)	
**Height z-score**			0.393
< -2	12	3	
-2–0	20 (51)	3	
> 0	7	5 (45)	

### 2. National survey

Thirty-four surveys regarding cleft lip and cleft palate that were performed in Japan were reviewed. Of the 16,452 patients from these 34 reports, 1,813 from 3 reports were studied in incidence surveys conducted at obstetrics facilities outside of Oita Prefecture, and 14,539 from the other 31 reports were investigated in surveys conducted at single centers, comprising 29 dental and oral surgery facilities and 2 plastic surgery facilities. In the 1,370,469 live births included in the incidence survey, the occurrences of CL in 580 patients (4.2 /10,000 births), CLP in 853 patients (6.2/10,000 births), and CP in 380 patients (2.8/10,000 births) were confirmed, indicating similar incidences of CLP and CP as in the regional survey. Data on 14,539 patients with cleft lip and cleft palate included in the 31 single-center surveys are shown in Table 5. Both CL and CLP occurred on the left side approximately twice as often as on the right side, and they were also approximately 1.5 times more commonly observed in boys than in girls. In contrast, the incidence of CP was 1.5-fold greater in girls than in boys. The occurrence of associated malformations was lowest in patients with CL and highest in patients with CP. The above clinical characteristics all tended to be similar to the findings from the regional survey; however, the percentage of patients with associated malformations was lower in the national survey than in the regional survey.

**Table 5:** Distribution of cleft pattern by cleft type, sex, and associated birth defects in a nationwide study.

	Cleft type					Sex		Associated	Total
	Unilateral		Bilateral	Median				birth defects	
	Left	Right				Male	Female	(%)	
**CL**	1725	917	315		8	1657	1308	9.8	2965
	(L:R = 1.9:1)					(M:F = 1.3:1)			
**CLP**	3079	1711	1972			4124	2638	18.8	6762
	(L:R = 1.8:1)					(M:F = 1.6:1)			
**CP**				Complete	3417	1890	2922	35.7	4812
				Incomplete	1395	(M:F = 1:1.5)			
								21.2	14539

Of the 14,539 patients from single-center surveys, detailed information on associated malformations in both CLP and CP could be confirmed in 1,231 patients, and the details and frequencies of the associated malformations were compared between these patients and the regional survey (Table 6). The percentage of patients with associated malformations was significantly greater in the regional survey conducted at NICU facilities, and the percentages were especially high for congenital heart disease and chromosomal abnormalities. Such characteristic findings were similarly observed when comparisons were made between the incidence survey conducted at obstetrics and gynecology facilities and the regional survey (19.6% vs. 41.3% for some type of associated malformation, 4.2% vs. 21.7% for congenital heart disease, and 4.2% vs. 16.3% for chromosomal abnormalities).

**Table 6:** Associated congenital malformations in nationwide and regional studies.

	Nationwide study	Regional study	P Value
	(CLP 675, CP 556)	(CLP 63, CP 29)	
	n = 1231 (%)*	n = 92 (%)*	
**Nervous system**	12 (1.0)	3 (3.3)	0.080
**Eye, ear, face, and neck**	44 (3.6)	2 (2.2)	0.766
**Circulatory system**	84 (6.8)	20 (21.7)	< 0.001
**Respiratory system**	7 (0.6)	1 (1.1)	0.439
**Other digestive system**	15 (1.0)	2 (2.2)	0.333
**Genital organs**	7 (0.6)	4 (4.3)	0.005
**Urinary system**	7 (0.6)	2 (2.2)	0.125
**Musculoskeletal system**	45 (3.7)	6 (6.5)	0.161
**Other congenital malformation**	47 (3.8)	8 (8.7)	0.050
**Chromosomal abnormality**	6 (0.5)	15 (16.3)	< 0.001
**Any birth defects**	244 (19.8)	38 (41.3)	< 0.001

The nationwide study investigated 29 oral surgery facilities and 2 plastic surgery facilities; The regional study investigated 5 NICU facilities.

* Each applicable type of birth defect was counted (multiples items allowed per patient).

## Discussion

In Japan, nearly all neonates with CLP and CP are transferred to the nearby NICU early after birth to receive multidisciplinary management. There, an accurate diagnosis of associated malformations is performed. On the other hand, neonates with CL are sometimes not transferred to the NICU if they do not show a feeding disorder or other issues. These patients were therefore excluded from this survey. Based on these facts, we postulated that the total occurrence of CLP and CL within a specific region can be ascertained, and that associated malformations can be accurately evaluated by conducting an epidemiological survey concerning CLP and CL at NICU facilities, and the present regional survey was conducted. In addition, our national survey includes the greatest number of patients among all previous epidemiological surveys that investigated cleft lip and cleft palate in Japan.

The incidence of cleft lip and cleft palate, cleft laterality, and the male-to-female ratio in Japan were similar to the results from international epidemiological surveys.[1,4,5] With the improvements in the resolution of ultrasound equipment and the technical skills of ultrasonographers, an enhancement in the rate of prenatal diagnosis has been observed globally.[6–8] Contrary to previous reports that demonstrated the rate of prenatal CL diagnosis to be 18–48%, the rate of prenatal diagnosis exceeded 50% in the present study.[7,8] On the other hand, the rate of prenatal diagnosis of CP alone has been reported to be only about 2%, similar to the present study, indicating the difficulty in making the diagnosis of CP prenatally.[8] Non-invasive prenatal testing has become available in Japan since 2013, and it has been shown in a study of 7,740 pregnant women tested that, of the 111 women who had positive test results and were confirmed with invasive tests to carry a fetus with chromosomal aneuploidy, 110 opted to terminate their pregnancy.[9] With the widespread use of such prenatal diagnostic testing in the future, it is possible that the incidence of symptomatic cleft lip and cleft palate may decline.

Although the occurrence of associated malformations was similar between the regional survey in the present study and international surveys, this occurrence was lower in the present national survey than in international surveys.[10,11] This finding was thought to be due to the fact that previous surveys in Japan investigated dental and oral surgery or obstetrics facilities. In other words, severely ill patients who die during infancy, such as those included in the present regional survey, do not receive treatment at dental and oral surgery or plastic surgery facilities. Furthermore, surveys involving obstetrics facilities resulted in lower than actual occurrences of each type of associated malformation, because congenital heart disease and chromosomal abnormalities are not accurately diagnosed in such surveys. Regarding different types of associated malformations, compared to a European epidemiological survey, the present regional survey resulted in a greater occurrence of congenital heart disease and chromosomal abnormalities, but a similar occurrence of all other associated malformations.[10] The regional survey in the present study investigated patients admitted to the NICU where detailed examinations are thoroughly performed, and this was considered to be the reason why the rate of diagnosing these two disorders was higher.

About half of the patients in the regional survey used a Hotz plate, which has been widely used preoperatively as a dynamic assistive tool for the purpose of encouraging maxillary growth and providing feeding support in patients with CLP or CP in Japan.[12] However, sufficient evidence that demonstrate the long-term effectiveness of assistive tools is still lacking.[13] It has been reported that tube feeding is often required when there is an associated malformation, and, similarly in the present survey, a large percentage of patients with CP who commonly have associated malformations required tube feeding.[14]

There is controversy with regards to the appropriate procedure and timing of cleft palate repair, and the medical management approach differs depending on the country. For example, in Japan, it is common to use a one-stage cleft palate repair in which simultaneous closure of the soft and hard palate cleft is performed around 18 months of age taking into account language development and maxillofacial growth.[4,15] The timing at which patients undergo cleft palate repair is delayed when a cardiorespiratory disorder or growth disorder caused by an associated malformation is observed.

It has been reported in patients with cleft lip and cleft palate that various types of conditions arise after cleft palate repair. These conditions include malocclusion (82%), serous otitis media (71%), dysphonia due to hypernasality (37%), postoperative feeding disorder (21%), and hearing loss (7%), indicating the necessity of long-term management involving a multidisciplinary approach.[4,14,16–19] With regards to the long-term physical development of patients with cleft lip and cleft palate, Prahl et al. performed physical measurements of CLP patients at 14 months of age and found that the z-scores for body weight and height were -0.72±0.77 and -0.37±0.78, respectively. [20] Their results, similar to the present study findings, showed that the patients maintained physical development that is similar to that of the general population.

There are several limitations to this study. The first is that, because the regional survey was conducted in NICU facilities, patients with CL were inevitably excluded from the survey. The second limitation is that the incidence of cleft lip and cleft palate may change in the future due to the increasing mean maternal age and the availability of next-generation prenatal diagnostic testing in Japan. In order to ascertain accurate epidemiological data, it is necessary to conduct surveys at an appropriate time period in accordance with the changes in domestic childbirth status or in prenatal diagnostic methods.

## Conclusions

The incidences of cleft lip and cleft palate in both a specific region and nationwide in Japan were similar to those reported in international epidemiological surveys. An epidemiological survey on the overall incidence of cleft lip and cleft palate is possible by conducting a survey of obstetrics facilities. In contrast, for malformations associated with cleft lip and cleft palate, accurate information can be obtained by conducting a survey of patients admitted to the NICU.

## Abbreviations

CL: cleft lip
CLP: cleft lip and palate
CP: cleft palate
NICU: neonatal intensive care unit
